# Retraction Note: Sulforaphane Improves Ischemia-Induced Detrusor Overactivity by Downregulating the Enhancement of Associated Endoplasmic Reticulum Stress, Autophagy, and Apoptosis in Rat Bladder

**DOI:** 10.1038/s41598-023-34470-8

**Published:** 2023-05-03

**Authors:** Huai-Ching Tai, Shiu-Dong Chung, Chiang-Ting Chien, Hong-Jeng Yu

**Affiliations:** 1 Department of Urology, National Taiwan University Hospital and National Taiwan University College of Medicine, Taipei, Taiwan; 2 Graduate Institution of Clinical Medicine, National Taiwan University College of Medicine, Taipei, Taiwan; 3 Department of Urology, Far East Memory Hospital, New Taipei City, Taiwan; 4 Graduate Program in Biomedical Informatics, College of Informatics, Yuan-Ze University, Chung-Li, Tao-Yuan, Taiwan; 5 Department of Life Science, National Taiwan Normal University, Taipei, Taiwan

Retraction of: *Scientific Reports* 10.1038/srep36110, published online 08 November 2016

Editors have retracted this Article.

After publication it was brought to Editors attention that two panels in Figure 5 were previously used in older publications from some of the same authors^1, 2^. The panel 2WBI group, 3-NT in Figure 5 seems very similar to the SP group, CD68 presented in Figure 4 in^1^ and the panel 4WBI group, 3-NT in Figure 5 seems very similar to the SP group, CD68 shown in Figure 1E in^2^.

Editors requested original raw data underlying the paper, but the Authors were not able to provide all of the data, nor did the data provided contained sufficiently detailed metadata to allow for verification of its veracity. The Editors therefore not longer have confidence in the reliability of the data presented in this Article.

None of the Authors have responded to correspondence from the Editors about this retraction.
